# Development and functions of the area opaca of the chick embryo

**DOI:** 10.1016/j.ydbio.2024.12.002

**Published:** 2025-03

**Authors:** Hyung Chul Lee, Yara Fadaili, Claudio D. Stern

**Affiliations:** a School of Biological Sciences and Technology, College of Natural Sciences, Chonnam National University, 77 Yongbong-ro, Gwangju, 61186, South Korea; b Department of Cell and Developmental Biology, University College London, Gower Street, London, WC1E 6BT, UK

**Keywords:** Primitive streak, Induction, Marginal zone, Epiboly, Tissue fusion, Extraembryonic tissues

## Abstract

Before radial symmetry-breaking of the blastoderm, the chick embryo is distinctly divided into a central area pellucida and a surrounding region, the area opaca. In this review, we focus on the area opaca and its functions. First, we survey current knowledge about how the area opaca is formed during the intrauterine period and how it sets up its initial tissue structure. With the formation of a vascularized mesoderm layer, the area opaca becomes subdivided into an inner area vasculosa and an outer area vitellina, which contribute to the development of extraembryonic membranes: the yolk sac and chorion. Second, we review the various functions of the area opaca during development including supplying nutrients, driving the expansion of the embryo by a specialized population of edge cells, and active, instructive signaling that plays a role in the establishment of embryonic polarity and orchestrates the formation of another extraembryonic tissue, the marginal zone, essential for positioning the first midline structure, the primitive streak, at the beginning of gastrulation.

## Highlights

• The chick embryo is divided into a central area pellucida and a peripheral area opaca. Here we focus on the latter.
• We review current knowledge about how the area opaca is formed.
• The area opaca then vascularizes, becoming subdivided into an inner area vasculosa and an outer area vitellina.
• The area opaca contributes to the development of extraembryonic membranes: the yolk sac and chorion.
• We review the functions of the area opaca during development including:
  o supplying nutrients,
  o driving the expansion of the embryo by a specialized population of edge cells,
  o active, instructive signaling that plays a role in the establishment of embryonic polarity and orchestrates the formation of another extraembryonic tissue, the marginal zone, essential for positioning the first midline structure, the primitive streak, at the beginning of gastrulation.
• We present a list of molecular markers that characterize the area opaca.

## Graphical abstract

**Figure:**
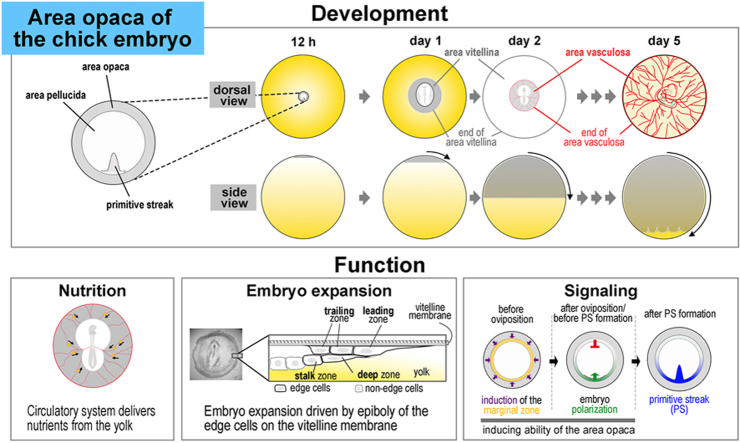

## 1 Tissue structure and cellular properties of the area opaca

### 1.1 Formation

**Fig. 1:**
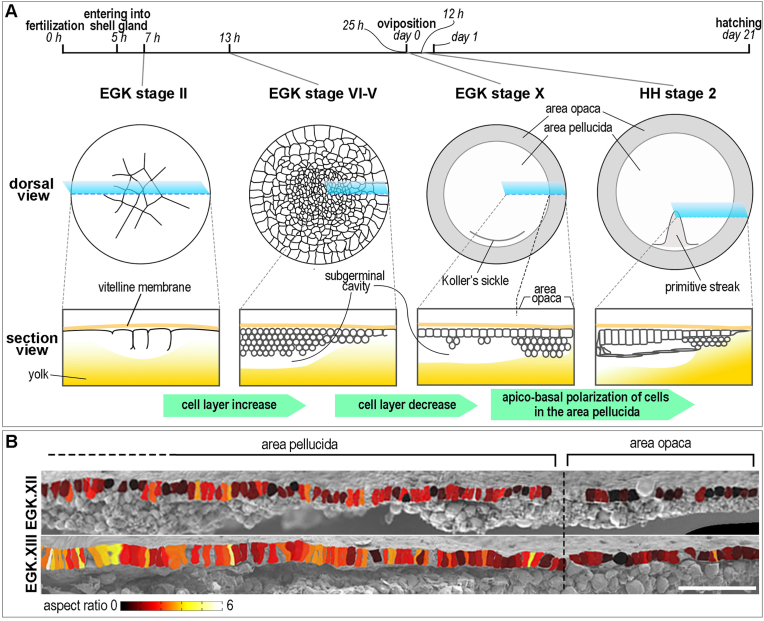
**Formation and cellular changes in the area opaca.** (A) Formation of the area opaca during pre-primitive-streak stages. The area pellucida and the area opaca arise at around EGK stage VII, with a cell layer decrease in the central but not in the peripheral region of the blastoderm. (B) Area pellucida-restricted cell polarization during primitive streak formation. The aspect ratio of epiblast cells was measured from scanning electron micrographs and is represented as a heat map. Scale bar: 100 μm. (B) reproduced from Fig. 2 B, C of (Lee et al., 2020).

In the chick, fertilization occurs in the infundibulum of the mother; the zygote travels along the oviduct towards the shell gland (the uterus) without cell division. The first and second cleavages occur at around the time when the fertilized egg reaches the shell gland (Eyal-Giladi and Kochav, 1976; Lee et al., 2013). The fertilized egg undergoes continuous cleavage in the shell gland, spending around 20 h before finally forming a disc-shaped, single-layered structure called blastoderm, which comprises 20,000–50,000 cells at the time of oviposition (egg laying) (Spratt and Haas, 1960). Until Eyal-Giladi & Kochav (EGK) (Eyal-Giladi and Kochav, 1976) stage IV (∼6h in the shell gland), cleavages occur primarily in the central region but are delayed in the periphery of the embryo, resulting in the formation of smaller cells in the center compared to the periphery of the blastoderm (Fig. 1A) (Bellairs et al., 1978; Eyal-Giladi and Kochav, 1976; Lee et al., 2013; Nagai et al., 2015). The peripheral cells become smaller by cleavage events after EGK stage V (8h and over) (Eyal-Giladi and Kochav, 1976; Lee et al., 2013). During subsequent development, the central region of the blastoderm reduces its number of cell layers from around five to one, beginning around EGK stage VI, in a direction presaging the future posterior-to-anterior axis (Eyal-Giladi and Kochav, 1976; Lee et al., 2013). This layer reduction causes the embryo to become divided into two distinct regions, the area pellucida (meaning ‘transparent’, inner circular region) and the area opaca (meaning ‘non-transparent’, outer marginal region) (Fig. 1A) (Eyal-Giladi and Kochav, 1976). Although limited access to the embryo at these stages has hampered studies on the molecular underpinnings of these processes, a few recent studies have explored the accompanying cellular events (Nagai et al., 2015) and conducted transcriptomic analyses (Hwang et al., 2018). All the early embryonic structures including the primitive streak, the neural plate, and the whole embryonic axis including somites form in the area pellucida, while the cells from the area opaca do not contribute to early embryonic structures and do not show embryo-forming potency when cultured in isolation from the area pellucida (Khaner et al., 1985).

### 1.2 Development of the area opaca

#### 1.2.1 Structure of the area opaca

After egg laying when the embryo is at around EGK stage X, the area opaca is multi-layered, with deeper cells in contact with the underlying yolk. In contrast, the area pellucida has a single-layered epiblast underlain by “islands” of hypoblast cells (about 5–20 cells each), and separated from the yolk by the subgerminal cavity (Fig. 1A) (Eyal-Giladi and Kochav, 1976). Cells in the deep layers under the epiblast of the area opaca are called ‘germ wall’ (Andries et al., 1983; Vakaet, 1970). Initially, epiblast cells in both areas have cuboidal shape, then only cells of the area pellucida become gradually elongated along the apical-basal axis, acquiring a columnar shape and expressing polarity markers such as PKCζ, RAC1, and RHOA with gradually increasing intensity and progressive localization (Fig. 1A–B) (Lee et al., 2020; Voiculescu et al., 2014). On the other hand, the cells of the area opaca maintain their cuboidal shape before gastrulation and then become flattened during epiboly, through which the embryo expands and grows (Fig. 1A–B) (Bellairs, 1963; Downie, 1976; Downie and Pegrum, 1971; Lee et al., 2020). The endodermal cells of the area opaca contain yolk droplets in the cytosol, which are much larger than those in the overlying ectodermal cells or in the epiblast of the area pellucida (Bellairs, 1958, 1963). When the embryo starts to expand at around 10 h' incubation from freshly laid eggs (approximately Hamburger & Hamilton stage 2) (HH) (Hamburger and Hamilton, 1951), the cells making up the most peripheral thin (2–4 cells thick) region of the area opaca, called the margin of overgrowth (Patterson, 1909) or simply “edge cells” (Bellairs et al., 1969; Downie and Pegrum, 1971; Lee et al., 2022a), change their morphology and acquire very long (40–60 μm) cellular processes, projecting outwards. The edge cells are the only cells that attach to the overlying vitelline membrane and migrate outwards, using these cellular projections, until they enclose the entire yolk mass by day 4–5 (New, 1959).

#### 1.2.2 Area vitellina and area vasculosa

**Fig. 2:**
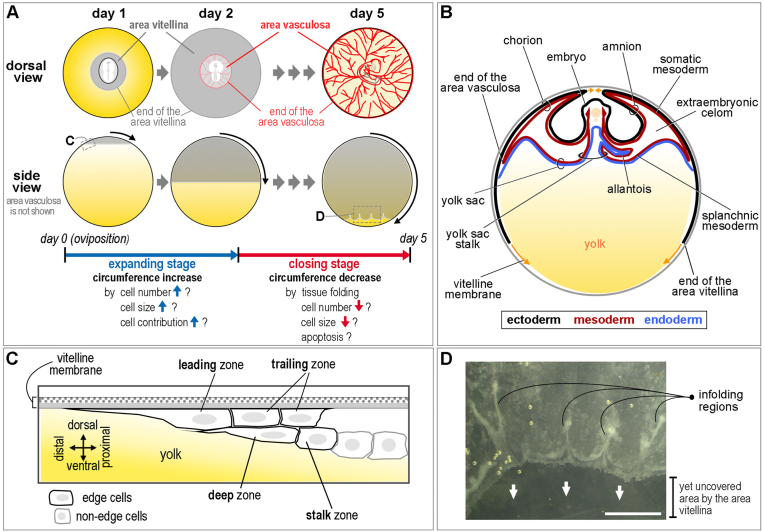
**Development of the area opaca and the epiboly.** (A) Development of the area opaca during expansion of the embryo over the yolk mass. During the expanding stage, the edges of the area vitellina move to reach the equator of the yolk on day 2. It reaches the south pole of the yolk during the closing stage on day 5. The expansion of the area vasculosa by invasion of the vascularized mesodermal layer follows the expansion of the embryo, reaching the equator on day 5. Possible cellular mechanisms for increasing and decreasing the circumference are indicated. (B) Formation of extraembryonic membranes in a developing embryo at day 3. Ectoderm, mesoderm and endoderm are marked in black, red and blue, respectively. The opposing arrows indicate the closing of the amnion (upper) and the migration of edge cells of the embryo (lower). (C) Schematic diagram of the edge region of the embryo (the edge of the area vitellina). (C) is reproduced from Fig. 1B of (Lee et al., 2022a). (D) The edge region at the end of the closing stage showing tissue infoldings. Arrows indicate the direction of closing. Scale bar: 3 mm. (For interpretation of the references to colour in this figure legend, the reader is referred to the Web version of this article.)

The area opaca, which comprises an ectodermal layer above and a dispersed yolky endodermal layer below at primitive streak stages, is gradually invaded by the mesodermal layer (between the previous two) and becomes vascularized from day 2 (Fig. 2A) (Bellairs, 1963; Sheng, 2010). This region of the area opaca is called “area vasculosa” due to its prominent vascularization of the invading mesodermal layer, whereas the surrounding area opaca, except edge cells, unoccupied by mesoderm is called the “area vitellina" (Fig. 2A–B) (Patten, 1971; Romanoff, 1960; Sheng, 2010). The distal end of the area vasculosa is demarcated by a thick circumferential extraembryonic blood vessel, called the “sinus terminalis”. The pattern of major blood vessels in this extraembryonic region is largely constant between embryos, suggesting that it is regulated by robust patterning mechanisms, to ensure that the embryo has efficient access to its environment and thus, to nutrients and gases. However, the nature of these patterning mechanisms is not yet known. There is a time gap between the expansion of the area vitellina and that of the area vasculosa. When the free edge of the embryo reaches the equator of the egg at day 2, the area vasculosa occupies only a small area. By day 4–5, the area vitellina covers the whole egg yolk, and the area vasculosa reaches the equator (Fig. 2A). By day 14–15, the area vasculosa covers the whole egg yolk at the expense of the area vitellina (Grodzinski, 1934; Mayer and Packard, 1978). The ectoderm of the area opaca including the area vitellina and area vasculosa comprises a single layer, at the head process stage (∼18h, stage HH5), then becomes two or more layers after 2–3 days (Bellairs, 1963). The extraembryonic mesoderm is known to be derived from the primitive streak, especially from its posterior part (Psychoyos and Stern, 1996; Sawada and Aoyama, 1999; Spratt, 1946); however, the possibility that some of this mesoderm receives contributions from other tissues, such as the hypoblast, endoblast or endoderm, has not yet been investigated.

The expansion of the mesoderm of the area vasculosa is a locally autonomous process, as it still continues even when expansion of the blastoderm itself is inhibited, or when the central embryonic region is removed (Mayer and Packard, 1978). This expansion is driven by specialized cells located distally near the sinus terminalis, exhibiting morphology of actively migrating cells such as a leading edge with numerous filopodia (Flamme, 1987; Mayer and Packard, 1978). Local application of cytochalasin D, an inhibitor of actin polymerization, selectively blocks migration of the extraembryonic mesoderm (Flamme, 1987).

#### 1.2.3 Extraembryonic membranes derived from the area opaca

There are four extraembryonic membranes: yolk sac, amnion, chorion, and allantois; the area opaca contributes to the yolk sac and chorion. During expansion of the extraembryonic mesoderm as it invades the space between the ectoderm and the endoderm of the area opaca, this mesoderm splits into two layers, somatic (adjacent to the ectoderm) and splanchnic (adjacent to the endoderm), defining a cavity, the extraembryonic celom, between them (Fig. 2B) (Bellairs, 1963). The yolk sac is made up of extraembryonic endoderm. Initially this is associated with the adjacent extraembryonic ectoderm, but later, as the mesoderm invades and splits, the yolk sac endoderm is associated with the deeper layer, splanchnic mesoderm. The yolk sac stalk, which connects the yolk sac endoderm to the lumen of the embryonic gut (Fig. 2B), is at least partly derived from the hypoblast, which covers the central area pellucida by EGK XIII (Stern and Downs, 2012). From day 2 when the area vasculosa actively produces blood cells and vessels, the yolk sac begins to function to take up yolk materials (Flamme, 1987). The amnion starts to appear at ∼30h and encloses the embryo after 3 days of incubation, generating the fluid-filled amniotic cavity (Fig. 2B) (Romanoff, 1960). As the amnion and the chorion (the outermost extraembryonic membrane) develop together, they both comprise ectoderm and somatic mesoderm, but in a different order: ectoderm on the inner side and mesoderm on the outer side in the amnion, and vice-versa in the chorion (Fig. 2B) (Romanoff, 1960). The two membranes are separated by extraembryonic celom and protect the developing embryo from external shock. Unlike other extraembryonic membranes, the allantois is derived from the hindgut, an intraembryonic region from day 3 (Fig. 2B) (Leeson and Leeson, 1963; Romanoff, 1960). After rapid growth, it fuses with the chorion to become the chorioallantois (Romanoff, 1960). The allantois functions for collecting waste, supplying oxygen, and absorption of albumen. The double layer of mesoderm (chorion + allantois) develops a rich vascular network that is connected with the embryonic circulation by the allantoic arteries and veins (Patten, 1971).

## 2 Functions in early development

### 2.1 A source of nutrition at early stages of the embryo

The large yolk mass in the egg contains a large variety of carbohydrates, lipids, proteins, vitamins, and minerals, and is a great source of nutrition for most of the period of embryonic development and even for several days after hatching in birds. During the final stage of oocyte growth within the hen's ovary, large amounts of macromolecules synthesized from the liver are deposited into oocytes by receptor-mediated endocytosis (Shen et al., 1993). For the first two days of incubation, nevertheless, early chick embryos do not depend on nutrients from the yolk mass for their development, as shown by normal development in vitro in the absence of yolk (New, 1959; Spratt, 1948). Cells of the area pellucida and area opaca contain yolk droplets in their cytoplasm and use them, perhaps via intracellular digestive enzymes, until the onset of blood circulation (Bellairs, 1963). Those intracellular yolk droplets are thought to be derived from extracellular yolk materials by phagocytosis, rather than receptor-mediated endocytosis, based on electron microscopic observations (Bellairs, 1963; Mobbs and McMillan, 1981). First, there are filamentous processes of the endodermal cells of the area opaca, protruding towards extracellular yolk droplets that are non-membranous. Also, all the intracellular yolk droplets have membranous structures suggesting an inversion of some portion of the cell membrane by phagocytosis (Bellairs, 1963). Consistent with this, the endodermal epithelial cells of the area vitellina lack membrane proteins, such as LRP2 (low density lipoprotein receptor-related protein 2), amnionless and cubilin, which are responsible for endocytosis of yolk droplets, suggesting that phagocytosis might be a main way to absorb yolk components (Bauer et al., 2013).

After the circulatory system starts to form, cells begin to run out of intracellular yolk droplets and start to be supported by nutrients from the outside yolk mass via the circulation. The yolk is covered by a rapidly expanding two-layered area opaca containing ectoderm and endoderm. The yolk-facing endoderm, called yolk sac endoderm, is a monolayered epithelium. Nutrients in the yolk are first taken up by the yolk sac endodermal cells (Mobbs and McMillan, 1981). Then, after the extraembryonic circulation starts, they enter into overlying vascular systems derived from splanchnic mesoderm containing vascular endothelial cells, smooth muscle cells, and blood cells (Nakazawa et al., 2006). The yolk sac endoderm not only takes up nutrients but also processes them, including breakdown and repackaging before being secreted into the extraembryonic circulation system (Nakazawa et al., 2011). As a result, the composition of proteins and lipids circulating in the embryo becomes dissimilar from these components in the yolk (Speake et al., 1998). Also, yolk sac endodermal cells express genes encoding enzymes involved in the modification and synthesis of carbohydrates, lipids, and proteins at high levels (Nakazawa et al., 2011).

### 2.2 Embryo expansion and epiboly

#### 2.2.1 Principles of chick epiboly

Of all the cells in the developing embryo, only the edge cells at the periphery of the area opaca are attached to the vitelline membrane and migrate outward until they cover the whole yolk mass, which takes over 5 days in the chick: at day 2, the free edge of the embryo reaches the equator (Fig. 2A–C). During the first few hours of incubation, there is not much expansion of the embryo; expansion becomes prominent after about 10 h’ incubation (Downie, 1976). This rapid (200–500 μm/h) expansion by migration of edge cells shows a 200-fold increase in size. The edge consists of squamous epithelial cells with an extremely flattened morphology, including their nuclei (Downie and Pegrum, 1971). Edge cells project cell protrusions like lamellipodia and filopodia, and use them for amoeboid movement by attaching to laminar proteins like fibronectin in the vitelline membrane (Fig. 2 C) (Lash et al., 1990). This migration of edge cells as a sheet is responsible for embryo expansion and growth. Why only edge cells out of all the cells in the blastoderm adhere to the vitelline membrane is unknown. Nevertheless, some features of the adhering property of edge cells have been reported (New, 1959). When an isolated piece of the blastoderm containing a free edge all around is cultured, only cells near the original edge region, but not other cells with free edges, adhere to the vitelline membrane, suggesting that the adhering property of edge cells is intrinsic and is not merely acquired by proximity of cells to a cell-free environment (New, 1959). Which parts of the area opaca other than the edge can become edge cells when isolated is as yet unknown. Also, the adhering property of edge cells is limited to the side of cells facing the vitelline membrane, indicating that edge cells are also different along the vertical plane (vitelline membrane-yolk axis) (New, 1959).

As the embryo expands, the circumference of the embryo greatly increases (as a function of the square of the expanding radius) and cellular activities such as cell proliferation, increase in cell size, cell contribution by migration, or a combination of these are likely to be involved, at least until the rim of the embryo reaches the equator of the yolk, after which the circumference of the edge starts to decrease as it migrates to the South pole (Fig. 2A). Other species undergo epiboly mainly by increasing cell size (stretching alone) as in the red flour beetle (Jain et al., 2020), or together with radial intercalation as in the zebrafish (Warga and Kimmel, 1990), without cell proliferation. Cell size increase is almost certainly insufficient in the chick embryo because the diameter of the egg yolk is approximately 3 cm, suggesting that other mechanisms must take part in chick epiboly. One study proposed that edge cells do not have any distinctive proliferative signatures, suggesting that adjacent distal cells close to the edge proliferate and contribute to the edge (Futterman et al., 2011). Non-proliferation of edge cells may be due to their strong migratory nature, which shares the same molecules (e.g. microtubules and other cytoskeleton) with the cell division machinery. It is still unclear whether distal cells contribute to the edge with or without cell proliferation, and how the cell population at the edge is maintained during embryo expansion. As it passes the equator of the yolk, the embryo needs to change strategy to start to reduce the circumference (‘closing stage’; Fig. 2A) compared to when it undergoes expansion. A subset of edge cells may drop out from the leading circumference of the edge by a ‘purse-string’ mechanism to contract (Martin et al., 1994), or it may undergo apoptosis to reduce the number of cells. One study revealed regular tissue infoldings derived from the edge region during the closing stage, which may be an important way to reduce the circumference (Fig. 2A–D) (Lee et al., 2022a). However, the precise closing mechanisms have barely been studied and therefore remain largely unknown.

#### 2.2.2 Molecular properties of edge cells

The edge region of the embryo shows some features of “partial epithelial-to-mesenchymal transition” (partial-EMT) (Futterman et al., 2011). An intermediate filament that marks mesenchymal cells, vimentin, is strongly expressed in edge cells while an extracellular matrix protein, laminin, a major component of epiblast basement membrane, is absent from edge cells (Futterman et al., 2011). Recently, using in situ hybridization, immunohistochemistry and live imaging, the morphological and molecular properties of edge cells, including several markers exclusively expressed in them have been reported (Lee et al., 2022a). The markers include dishevelled-binding antagonist of beta catenin 2 (DACT2) and dickkopf WNT signaling pathway inhibitor 4 (DKK1/4), suggesting possible functions of WNT signals for migration of epithelial cell sheet and/or cell adhesion to the vitelline membrane (Lee et al., 2022a). Based on the spatial expression of the markers in different edge cells and their characteristic morphology, it was proposed that the edge cell region should be divided into four distinct zones: leading, trailing, deep, and stalk zones, which act as separate entities (Fig. 2B) (Lee et al., 2022a).

#### 2.2.3 Generation of mechanical tension during embryo expansion

The developing early chick embryo is under mechanical tension, which can be easily demonstrated by its almost immediate retraction (even at room temperature) when the edges are freed from the vitelline membrane; this tension is highest around 20–24 h when the embryo reaches around 12 mm in diameter (Bellairs et al., 1967; Downie, 1976). Epibolic movement of edge cells generates tension across the embryo and this is required for embryo expansion (New, 1959). As the embryo expands to encompass the whole yolk mass, overall tension, as well as cell size, increases enormously and the epithelial cell sheet would finally tear unless there is an accompanying increase in cell number. Indeed, cell division in the embryo relieves increasing tension generated by embryo expansion: when the developing embryo is treated with aminopterin, an inhibitor of cell division, many spaces appear in the epithelial cell sheet (Bellairs, 1954). When the tension generated by embryo expansion is modified, normal embryo development is affected (Bellairs et al., 1967; Kucera and Burnand, 1987; Kunz et al., 2023), suggesting that mechanical tension is required for normal embryonic development. However, how mechanical tension links chemical signals to cells to exert cellular changes in behavior and/or gene expression is not yet clear.

### 2.3 Signaling functions and embryo polarity

#### 2.3.1 Embryo polarity and the marginal zone

**Fig. 3:**
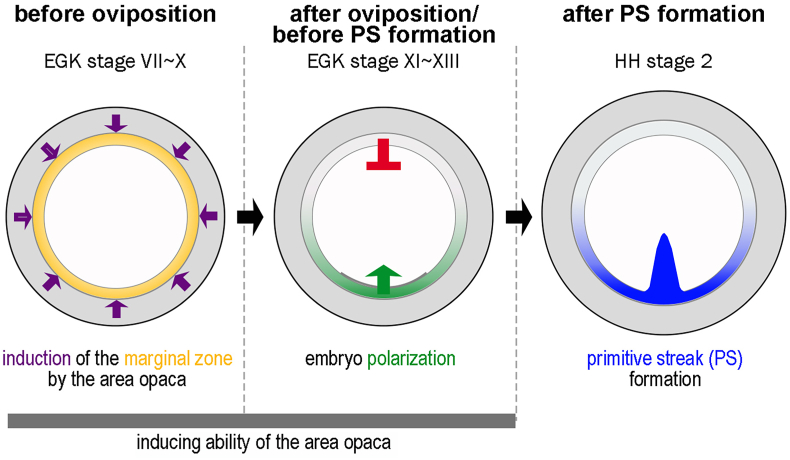
**A model for the role of the area opaca on embryo polarization and primitive streak formation.** Before oviposition, the area opaca induces the marginal zone property (posterior character) all the way around. A few hours after egg laying, one side of the marginal zone retains its posterior character, which becomes the signaling center to induce the primitive streak in the neighboring area pellucida. The inducing ability of the area opaca is lost once the primitive streak forms. Modified from fig. 5E in (Lee et al., 2022c).

Overt symmetry breaking of the chick embryo occurs with the formation of the primitive streak from one side of the area pellucida, which is dependent on receiving inductive signals from an adjacent extraembryonic tissue called the marginal zone (Fig. 3) (Bachvarova et al., 1998; Eyal-Giladi and Kochav, 1976; Eyal-Giladi and Spratt, 1965; Khaner and Eyal-Giladi, 1989; Lee et al., 2020; Torlopp et al., 2014). The marginal zone is the innermost ring of the extraembryonic epiblast between the opaca and the area pellucida (Bachvarova et al., 1998; Eyal-Giladi and Kochav, 1976; Lee et al., 2020). The marginal zone looks opaque like the area opaca due to its underlying endodermal layer, the germ wall, which is also made up of yolky cells, but is revealed as translucent when the germ wall is peeled off (Eyal-Giladi and Kochav, 1976). The posterior region of the marginal zone acts as a major signaling center for positioning the primitive streak and establishing embryo polarity. When a piece of the posterior marginal zone is grafted to another site of the marginal zone, an ectopic primitive streak is induced from the area pellucida adjacent to the grafted region (Bachvarova et al., 1998; Eyal-Giladi and Khaner, 1989; Eyal-Giladi and Spratt, 1965; Khaner and Eyal-Giladi, 1986; Spratt and Haas, 1960). The posterior marginal zone emits signals, including cVG1/GDF3 that collaborate with WNT signals to form the primitive streak (Khaner and Eyal-Giladi, 1986; Shah et al., 1997; Skromne and Stern, 2001, 2002). Nevertheless, the ability to induce a primitive streak is not restricted to the posterior region but seems to span the entire marginal zone (Bertocchini et al., 2004; Skromne and Stern, 2001; Spratt and Haas, 1960).

This raises some important questions. First, how do cells on one side of the marginal zone assess their position as “posterior” to establish embryo polarity? The ring-shaped marginal zone shows an opposite gradient of expression of cVG1 and bone morphogenetic protein 4 (BMP4) before the formation of the primitive streak (Bertocchini and Stern, 2012; Lee et al., 2022b; Shah et al., 1997) and marks the site where gastrulation will begin (near cVg1 expression and at the lowest point of the BMP gradient). cVG1 and BMP4 act as inducer and inhibitor, respectively. However, how cells decide the site of primitive streak formation at the region of highest expression of cVG1 and the lowest BMP4 activity has not been elucidated. A recent study suggests that cells in the marginal zone sense changes in morphogen status (inducers vs inhibitors) relative to their neighboring cells (‘Neighbourhood watch’ model) to position the site of primitive streak formation (Lee et al., 2022b). In support of this, counter-intuitively, when a sub-threshold amount of inducer is flanked by low amounts of inhibitor, expression of TBXT (a marker of the primitive streak) is generated (Lee et al., 2022b). These results suggest that cells establish their fates by assessing not the absolute levels of signals in their environment, but rather how these differ from the levels received by their neighbors. Another intriguing question, on a larger scale, is how positional information spreads to establish opposing gradients of morphogens across the embryo and consequently set embryo polarity. As the chick embryo at this stage is more than 3 mm in diameter, simple diffusion, which has a limited range (suggested to be limited to <100 cell diameters) (Crick, 1970; Kerszberg and Wolpert, 1998), cannot explain such long-range communication in a large embryo. Recently, it was shown that calcium firing activity propagates through the marginal zone via specific gap junctional communication (GJB2/6) to determine the site of primitive streak formation (Lee et al., 2024). The traveling calcium activity regulates and establishes a BMP gradient in the marginal zone via transcription factors such as NF-κB and NFAT (Lee et al., 2024). This calcium-based regulated network was proposed to inhibit the formation of an ectopic primitive streak, and can explain the low incidence of embryonic twinning (<5%) in normal development. This mechanism seems to be conserved across animal species including humans, and may provide a basis to explain how positional information is set up and maintained in large developing systems, where diffusion is an unrealistic mechanism (Lee et al., 2024).

#### 2.3.2 Possible roles of the area opaca on embryo polarity during normal development

The roles of the area opaca in providing nutrition from the yolk to the embryo and mechanical tension are well known. But whether the area opaca also has signaling roles in normal development has not been clear. Recently, a study revealed that the area opaca also plays three separate roles in the regulation of polarity of the early embryo (Lee et al., 2022c). First, grafting of the area opaca next to the area pellucida (i.e. ablation of the marginal zone) can generate a new marginal zone derived from the area pellucida epiblast. Second, when a piece of the area opaca is grafted next to an isolated anterior half-embryo fragment, the induced marginal zone has posterior character. Finally, a graft of area opaca from an early stage donor (EGK.X-XI, before primitive streak formation) to anterior embryo-fragment at late stage (Hamburger & Hamilton stage 2–3, after primitive streak formation) (Hamburger and Hamilton, 1951), by which time the ability of the host to generate new primitive streak has been lost, can rescue the ability of the anterior fragment to generate a new primitive streak (Lee et al., 2022c).

These recent studies suggest a possible signaling function of the area opaca to the marginal zone for normal development. Nevertheless, removal of the area opaca while leaving the marginal zone shows little or no effect on formation of the primitive streak in intact, pre-primitive-streak stage embryos (Khaner et al., 1985). These results suggest two possibilities for the signaling function of the area opaca. Either its role is redundant and not essential for normal development, or it may function at much earlier developmental stages such as intrauterine stages (Fig. 3). For the latter, it can be speculated that the area opaca may induce the marginal zone property by emitting secreting molecules, and subsequently, the marginal zone becomes polarized to retain its posterior character on one side and to set up embryo polarity (Lee et al., 2022c) (Fig. 3). Consistent with this idea, the area opaca strongly expresses the WNT ligand WNT8C, which also shows graded expression in the marginal zone (Lee et al., 2020; Skromne and Stern, 2001). Indeed, recent findings from our laboratory suggest that upregulation of WNT signaling using a potent GSK3β inhibitor, 6-Bromoindirubin-3′-oxime (BIO), can mimic the inducing ability of the area opaca, whereas the WNT inhibitor 4-(1,3,3a,4,7,7a-Hexahydro-1,3-dioxo-4,7-methano-2H-isoindol-2-yl)-N-8-quinolinyl-Benzamide (IWR-1) impairs this induction as well as the maintenance of the marginal zone in intact embryos (Fadaili et al., 2024). Additionally, the area opaca loses its inducing ability after primitive streak formation (Lee et al., 2022c) (Fig. 3). In line with this, *WNT8C* expression in the area opaca decreases by HH stage 2 when the primitive streak forms, while expression of its inhibitor, *DKK1* seems to increase in the same region. These data suggest that one or more WNT ligands activating the canonical pathway are the key marginal-zone inducing signals emanating from the area opaca.

### 2.4 Gene expression in the area opaca

**Fig. 4:**
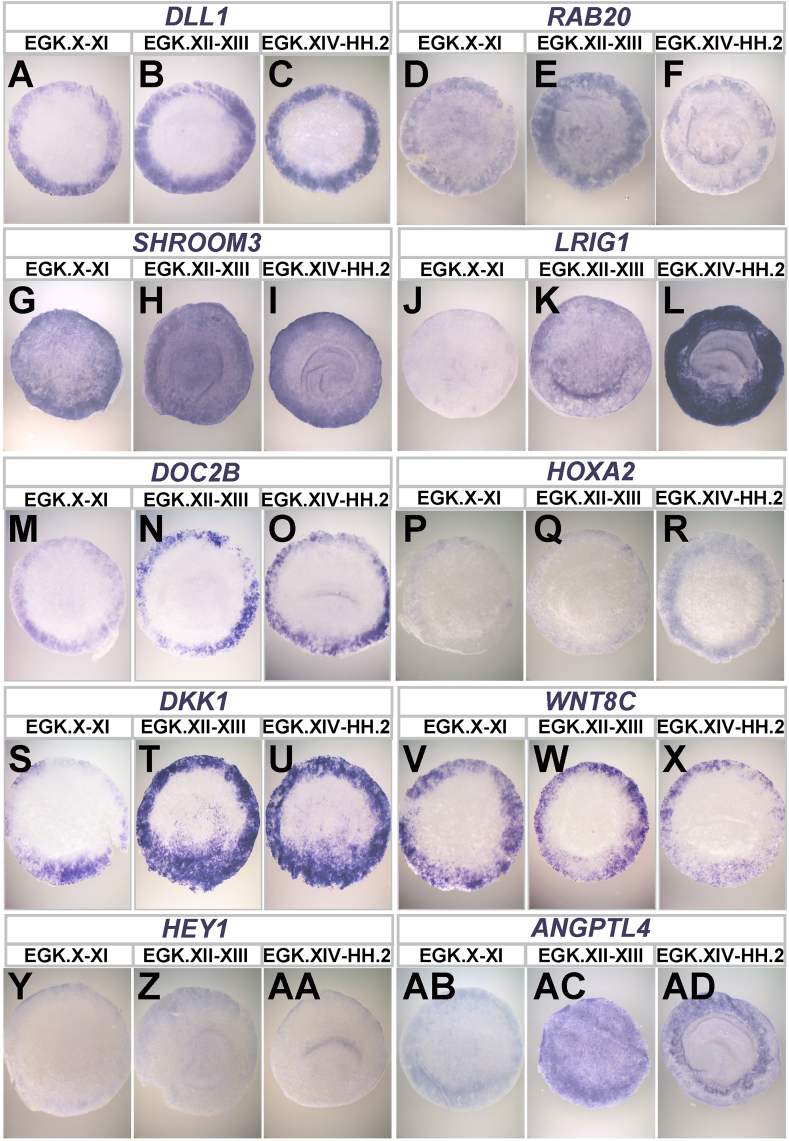
**Expression of selected marker genes enriched in the area opaca** (see Table 1). Expression patterns of genes as visualized by in situ hybridization at three stages: EGK X–XI, EGK XII–XIII, and EGK XIV–HH2. Embryos are shown anterior side up. Note that VLDLR expression is undetectable by in situ hybridization. The original data were reported previously (Lee et al., 2020).

Regarding gene expression profile, the area opaca of pre-primitive-streak stage embryos has a distinct profile compared to the marginal zone and the area pellucida, while it is closer to that of the former (Lee et al., 2020). Table 1 and Fig. 4 provide a list of genes enriched in the area opaca and their expression patterns, as revealed by RNA sequencing and mRNA in situ hybridization respectively, derived from a previous study (Lee et al., 2020). For all such genes enriched in the area opaca, the endodermal layer (germ wall) shows higher levels of expression level than the overlying epiblast (Lee et al., 2020), raising the possibility that the germ wall has active roles that have not yet been elucidated.

**Table 1:** List of genes enriched in the area opaca at pre-primitive-streak stages (EGK stage XII-XIII).

Gene Symbol	Full Name	NCBI Gene ID
DLL1	delta like canonical Notch ligand 1	395820
RAB20	member RAS oncogene family	418753
SHROOM3 (ChEST243a9)	shroom family member 3	422636
LRIG1	leucine rich repeats and immunoglobulin like domains 1	427592
DOC2B	double C2 domain beta	417624
VLDLR	very low density lipoprotein receptor	396154
HOXA2	homeobox A2	396055
DKK4 (DKK1)	dickkopf WNT signaling pathway inhibitor 4	374156
WNT8A (cWNT8C)	Wnt family member 8A	396543
HEY1	hes related family bHLH transcription factor with YRPW motif 1	428365
ANGPTL4	angiopoietin like 4	769087

## 3 Conclusions

This review summarizes the formation and development of the first extraembryonic tissue in the chick, the area opaca as well as its versatile functions on embryo development. Despite some differences in the formation of extraembryonic tissues compared to other amniotes the relative timing of formation, overall architecture, and possible signaling functions of the area opaca that is similar to its mammalian counterpart (e.g. trophectoderm in mice and humans), provide important insight and sources to study extraembryonic tissues in an evolutionary context. Also, although conventional views limited the roles of the area opaca to nutrition and embryo expansion, recent studies covered here suggest that the area opaca may have more active signaling roles in the initial formation of the embryo. Additionally, the chick embryo shows some distinct mechanisms of embryo expansion and epiboly due to its prominently larger size compared to most other species, offering an alternative research model to study tissue expansion and fusion (e.g. the closing stage).

## CRediT authorship contribution statement

**Hyung Chul Lee:** Writing – review & editing, Writing – original draft. **Yara Fadaili:** Writing – review & editing. **Claudio D. Stern:** Writing – review & editing, Funding acquisition.

## Funding

Our work related to this topic was supported by Global - Learning & Academic research institution for Masters, PhD students, and Postdocs (LAMP) Program of 10.13039/501100003725National Research Foundation of Korea (10.13039/100007431NRF) grant funded by the Ministry of Education (No. Rs-2024-00442775) to HCL, by a 10.13039/100010269Wellcome Trust Investigator Award (107055/Z/15/Z) and by 10.13039/501100000268BBSRC grants BB/R003432/1 and BB/K007742/1 to CDS. YF was supported by a scholarship from the Custodian of The Two Holy Mosques’ External Scholarship Program from the Ministry of Education of Saudi Arabia (grant no. SE-12267).

## Declaration of competing interest

The authors declare no competing interests.

## Data Availability

No data was used for the research described in the article.
